# Fluctuating Asymmetry Spotted Wing Drosophila (Diptera: Drosophilidae) Exposed to Sublethal Doses of Acetamiprid and Nicotine

**DOI:** 10.3390/insects15090681

**Published:** 2024-09-09

**Authors:** Anetta Lewandowska-Wosik, Ewa Małgorzata Chudzińska

**Affiliations:** Department of Genetic, Institute of Experimental Biology, Faculty of Biology, Adam Mickiewicz University in Poznan, 61-614 Poznan, Poland; evpell@amu.edu.pl

**Keywords:** *Drosophila suzukii*, fluctuating asymmetry, acetamiprid, nicotine, sublethal doses, multigenerational cultures

## Abstract

**Simple Summary:**

Long-term exposure to low concentrations of insecticides can cause several adverse effects, both on target insects and on other organisms. Low doses can also generate tolerance in surviving offspring. One simple method to detect the consequences of long-term exposure to residual toxins is to measure deviations from ideal body symmetry. Fluctuating asymmetry (FA) is evidence that insects were stressed during development. This study aimed to verify FA in the wing veins of *Drosophila suzukii*, a fruit pest controlled by the insecticide acetamiprid. To determine whether low doses of insecticide in the diet induce the asymmetry effect, multigenerational insect breeding was carried out on media supplemented with different concentrations of two insecticides. Nicotine was used as a positive control. Even in the first generation, low doses reduced fertility and caused vein asymmetry. This effect persisted in subsequent generations, indicating a lack of tolerance that led to complete insect death after 10 generations. *D. suzukii* proved extremely sensitive to acetamiprid, and FA is a good index of this sensitivity.

**Abstract:**

Long-term exposure to low concentrations of toxic substances can cause several adverse consequences ranging from molecular to morphological. Sublethal doses may also lead to increased tolerance in the offspring of surviving individuals. One of the consequences of such stress is deviations from the ideal body symmetry during development, reflected by increased levels of fluctuating asymmetry (FA). This research aimed to verify FA in the wing veins of insects belonging to the Drosophilidae family—*Drosophila suzukii*, a fruit pest controlled by the insecticide acetamiprid, a neonicotinoid. To determine whether FA varied depending on insecticides present in the diet, multigenerational cultures of *D. suzukii* were carried out on media supplemented with different concentrations (below the LC50) of two insecticides. Nicotine was used as a positive control. Fecundity decreased, the number of insects decreased, and breeding did not continue beyond the tenth generation. However, the FA level at different concentrations was similar, and high FA values were observed even at lower acetamiprid concentrations. We did not see significant changes in FA levels in subsequent generations. *D. suzukii* proved extremely sensitive to acetamiprid, and FA is a good index of this sensitivity.

## 1. Introduction

All organisms are constantly exposed to harmful substances present in the environment. The rapid development of agriculture obliges the production of a wide variety of pesticides, raising many questions regarding their safety and potential health and environmental risks. Often, pesticide residues that have been partially decomposed or diluted accumulate in soil, water, and plants in low doses [1,2,3]. In this way, they can directly or indirectly affect vertebrates and invertebrates. The best-known example of the negative impact of such substances on insects is pollinating insects. Bees can absorb pesticides from various sources, including food, direct contact with contaminated surfaces, inhalation, and exposure to water containing dissolved toxins. In most of these cases, insecticide concentrations are low, and their effects range from negligible to lethal [4,5,6,7,8,9,10].

One of the insects exposed to contact with insecticides at low concentrations is the vinegar fly, *Drosophila suzukii* (Matsumura, 1931), Diptera: Drosophilidae, also called the spotted wing Drosophila (SWD). It is a polyphagous fruit pest from Southeast Asia [11]. This species spread to Europe, Africa and Oceania [12]. This invasive species lays its eggs in intact fruits using its sclerotized ovipositor. Direct damage consists of larvae feeding on the pericarp, and secondary damage is caused by pathogens that penetrate the fruit, causing faster rotting and economic depreciation [13].

This pest is difficult to control, and many factors can expose its populations to sublethal doses of insecticides. One of these insecticides is acetamiprid (ACE (N-(6-chloropyridin-3-yl) methyl)-N′-cyano-N-methylethanimidamide), a neonicotinoid. Neonicotinoids are a class of insecticides that have a strong affinity for insect receptors, so it is believed that they are safe for other organisms [14]. Unfortunately, research shows that they can negatively affect other vertebrates, including mammals [14,15,16]. Neonicotinoids have a structure similar to nicotine; they are highly effective pesticides but are currently banned in Europe and the USA. (In this study, we used nicotine as a positive sample.) Like nicotine, neonicotinoids are agonists of nicotinic acetylcholine receptors in the nervous system. There is considerable concern about the bioavailability of neonicotinoids in the environment and the possible exposure of non-target organisms to insecticide residues, which have been detected at low concentrations in, e.g., pollen, nectar, soil, and water [17,18].

Sublethal effects are important for *D. suzukii* because its small size, feeding method, short development cycle, and use of different plant species as hosts mean that insecticides have a limited effect. Low doses of harmful substances can negatively affect the organism, especially in cases of long-term exposure [19,20]. This concerns various factors, such as survival, development, reproduction, learning ability, and behavior [4,21,22,23]. One method of determining whether chronic exposure to low concentrations of acetamiprid in food has negative effects is describing biomarkers of environmental stress such as fluctuating asymmetry (FA), a measure of developmental instability. The FA index reflects the degree of deviations between sides regarding the size of a given feature. FA is present when external stressors disrupt developmental processes that normally promote symmetrical growth. This is a non-directional, random, small deviation from perfect symmetry [24,25,26]. Among other insects, the emergence of FA under the influence of various factors has been demonstrated in *D. melanogaster* exposed to the antibiotic neomycin [27]; in *D. ananassae* as a result of nutritional stress [28]; and in *D. buzzatii* and *D. koepferae* raised on alternative breeding substrates [29]. Thus, we decided to determine to what extent it can be used to study *D. suzukii.* We aimed to test [i] whether exposing insects to sublethal doses of acetamiprid and nicotine in the diet causes deviations from wing symmetry; [ii] whether FA correlates with increasing concentrations of toxic substances; and [iii] whether there are FA changes in subsequent generations of insects compared with the F1 generation; in other words, can adaptation to these insecticides be observed?

## 2. Materials and Methods

### 2.1. SWD Colony and Experimental Design

A *D. suzukii* colony was established from wild individuals collected in 2019 from infested fruit growing (sweet cherries) near Wrzesnia in central Poland (N 52°18′56.931″ E 17°27′47.136″). The insects were reared on a modified sugar/yeast diet as described previously [30], in standard conditions: 23 ± 2 °C, under a 12:12 light-dark cycle, at 60% humidity. After optimizing rearing conditions, 5 virgin females and males were transferred to vials containing diet supplemented with sublethal toxin doses. After 72 h, the parent insects (P) were removed. The new, first generation of insects (definitely over 100 individuals in each bottle) was partly intended for analysis (30 individuals), and the rest was poured into new bottles with media so that the females would lay eggs and after 3 days poured out and disposed of. The new, second generation (three-day imago) was poured onto fresh diet with the tested substances, and the procedure was analogous until the tenth generation. Five replicates were performed for each concentration in seven independent experiments. Control diets without nicotine (N) and acetamiprid (A) were conducted in parallel. N and A were supplied by Sigma-Aldrich (St. Louis, MO, USA). To assess survival, emerged flies were counted on day 14 after oviposition. To calculate the percentage survival and sublethal doses in transgenerational studies, 50% survival percentage (SP50) was calculated as 50% of the total eggs that developed into adult flies. Dose-response tests were performed for acetamiprid and nicotine. To select insecticide doses for further analysis, acetamiprid was added to the medium at concentrations of 0.125, 0.25, 0.50, 0.875, and 1.0 μL/mL, and nicotine was added at concentrations of 0.05, 0.1, 0.15, 0.2, 0.3, 0.4, and 0.5 mg/mL. From the populations reared on standard medium, 100 *Drosophila suzukii* eggs were collected using a stereomicroscope and placed on a medium consisting of nicotine, acetamiprid, and standard medium. To assess survival, the number of hatched flies was determined. Three sublethal concentrations of acetamiprid were selected: 0.125, 0.250, and 0.500 µg/mL. We also used 0.875, which is above SP50 doses. Two concentrations of nicotine were used as positive controls (sublethal, 0.1 mg/mL; above SP50 doses, 0.2).

### 2.2. FA Measurement in the Wing Veins

**Figure 1:**
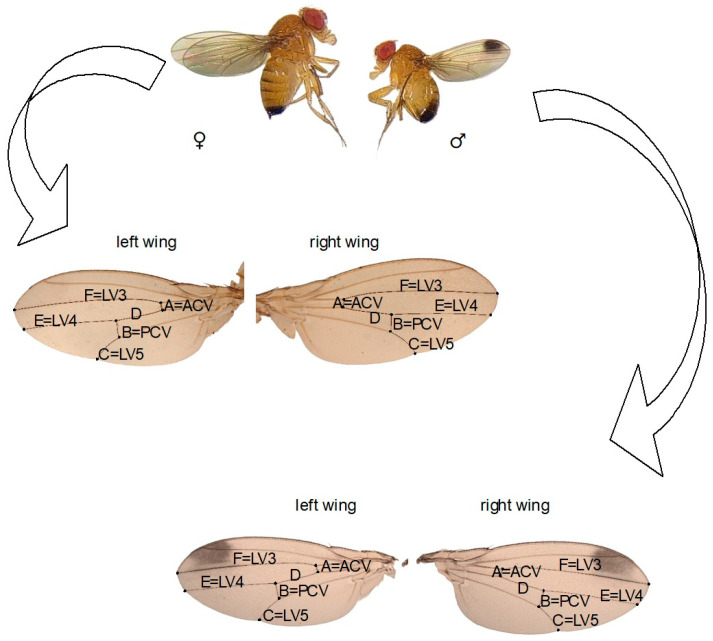
*Drosophila suzukii* adult. Right and left wings of a male and a female; from A to F, the measured veins are marked (LV 3,4,5 = longitudinal vein 3,4,5; PCV = posterior cross vein; ACV = anterior cross vein).

We randomly selected 15 female and 15 male *D. suzukii* flies (3 days old) reared on a diet with different concentrations of nicotine and acetamiprid from the first (F1), fifth (F5), and tenth (F10) generations. The insects were anesthetized using ether, and their wings were removed, placed on a slide, mounted in Euparal, and covered with a coverslip. Measurements of the veins of the left and right wings were taken under a microscope at 4× magnification using the CellSens software (Olympus Co., Tokyo, Japan). Six veins on the wing, marked A-F (Figure 1), were measured three times to reduce the risk of errors. FA was defined as the absolute difference between the veins of the right and left wings, standardized by the mean.

### 2.3. Statistical Analysis

Data were statistically analyzed using StatSoft Statistica (StatSoft Inc., Tulsa, OK, USA). The following statistics were calculated for vein length: arithmetic mean, minimum, maximum, standard deviation, and coefficient of variation. To determine the coefficient of FA, basic statistics, analysis of variance (ANOVA), the Shapiro–Wilk test, the Tukey test to check statistical significance, principal component analysis, and a dendrogram were calculated. Triplicate measurements of vein lengths were performed (90 measurements for each vein, A to F, from each of the six test concentrations plus the control, for generations F1, F5, and F10; F5—without A0.875 µg/mL, F10—without acetamiprid). The Kruskal–Wallis test determined the difference between triplicate wing measurements.

## 3. Results

The first results indicated that *D. suzukii* was so sensitive to acetamiprid that it could not be continued to the tenth generation, although this attempt was made three times. In generation F5, no adults survived at a concentration of 0.875. In generation F10, at concentrations of 0.125, 0.250, and 0.500, only two to five adults were recorded, so proper analyses could not be performed.

### 3.1. Vein Length

The absolute difference in the lengths of both veins yielded similar results, indicating high precision. Since there was no significant difference between replicates (*p* > 0.05), the data were combined. Acetamiprid and nicotine affected the average length of *D. suzukii* wing veins in F1. All veins except anterior cross-vein A were the longest in the control samples. In the remaining experiments, adding insecticides reduced vein length. No correlation was observed between the reduced length of the veins and the concentration of the tested toxic substances (Table 1).

### 3.2. Fluctuating Asymmetry

**Figure 2:**
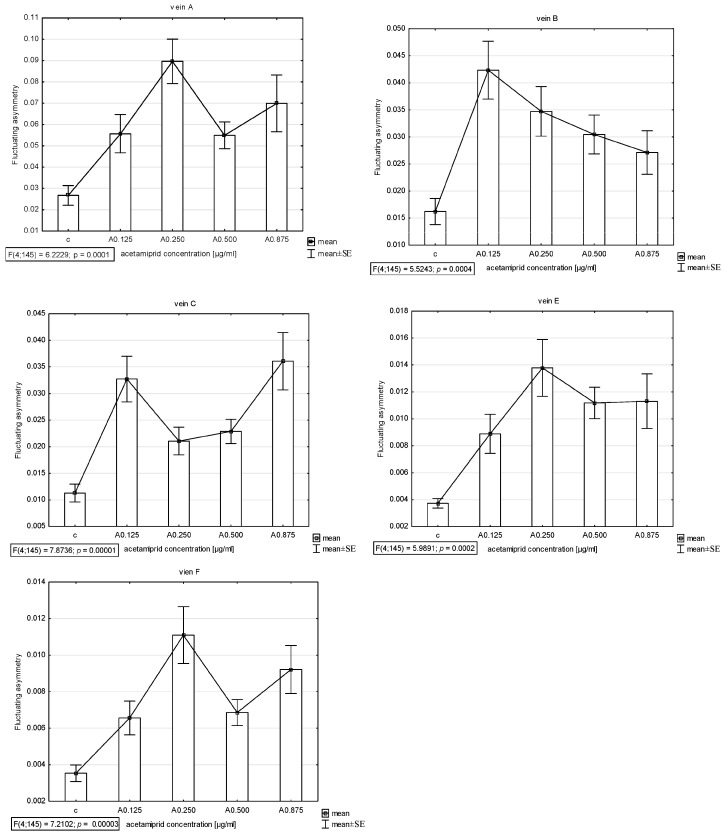
Graph of mean and standard error (SE) of fluctuating asymmetry for five studied veins (A, B, C, E, F) for four different concentrations of acetamiprid (0.125, 0.250, 0.500, and 0.875 µg/mL) and control. ANOVA (F) and *p*-value calculated.

**Figure 3:**
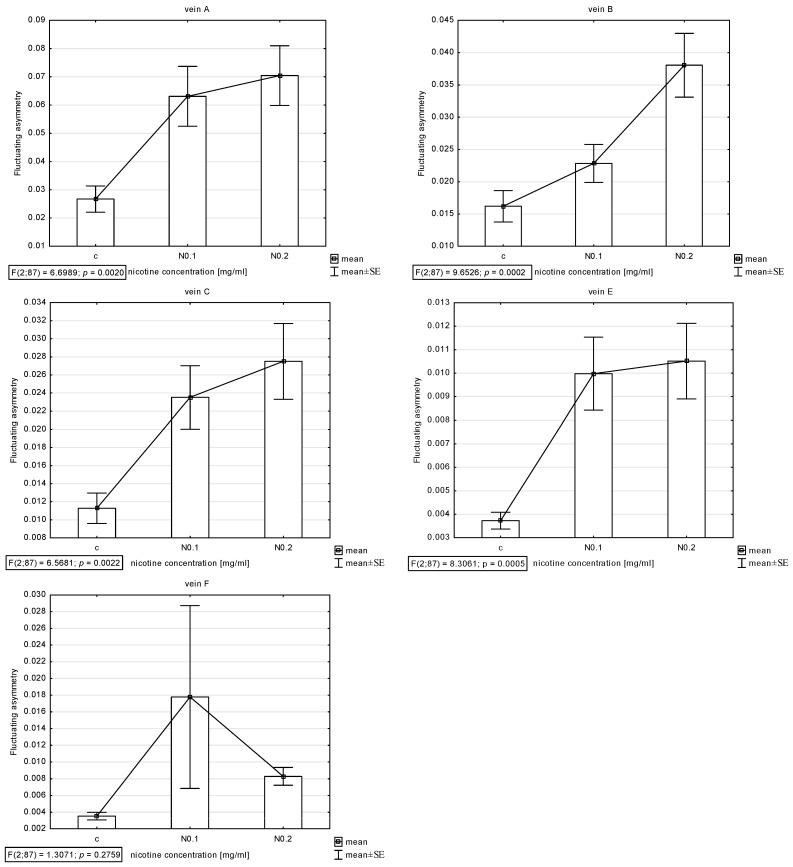
Graph of mean and standard error (SE) of fluctuating asymmetry for five studied veins (A, B, C, E, F) for two concentrations of nicotine (N: 0.1 and 0.2 mg/mL). ANOVA (F) and *p*-value calculated.

The presence of asymmetry was confirmed via an ANOVA examining the differences in length between longer and shorter veins in pairs in all veins (A, B, C, E, and F) except D, which was excluded from further analyses. The Shapiro–Wilk test showed that the FA index distribution was normal. Thus, the descriptive statistics were calculated. The highest FA for vein A was noted for acetamiprid at a concentration of 0.250 µg/mL and nicotine at a concentration of 0.2 mg/mL; for vein B, the highest FA was for acetamiprid at 0.125 µg/mL and nicotine at 0.2 mg/mL; for vein C, the highest was for acetamiprid at 0.875 µg/mL and nicotine at 0.2 mg/mL; for vein E, the highest was for acetamiprid at 0.250 µg/mL and nicotine at 0.2 mg/mL; for vein F, the highest was for acetamiprid at 0.250 µg/mL and nicotine at 0.1 mg/mL (Table 2; Figure 2 and Figure 3).

**Figure 4:**
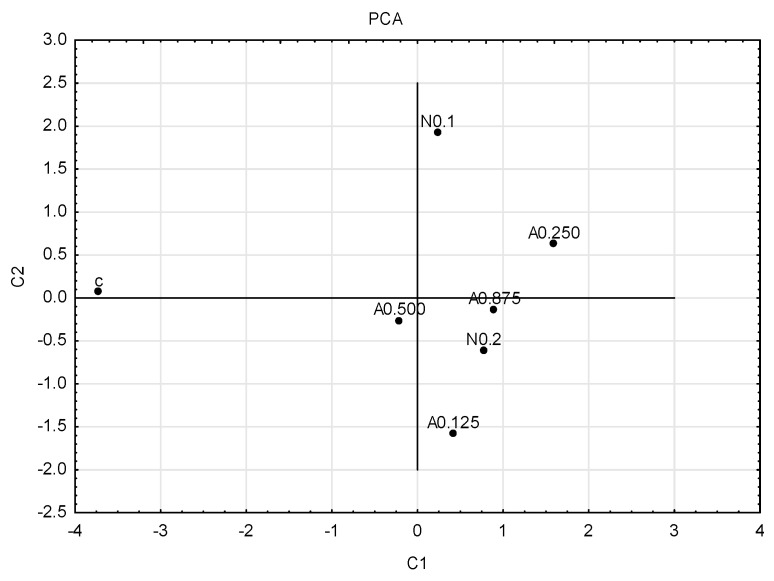
Graph of principal component analysis (PCA). First generation of insects; for four concentrations of acetamiprid (A = acetamiprid: 0.125, 0.250, 0.500, 0.875 µg/mL), for two concentrations of nicotine (N = nicotine: 0.1 and 0.2 mg/mL), and for the control (c).

**Figure 5:**
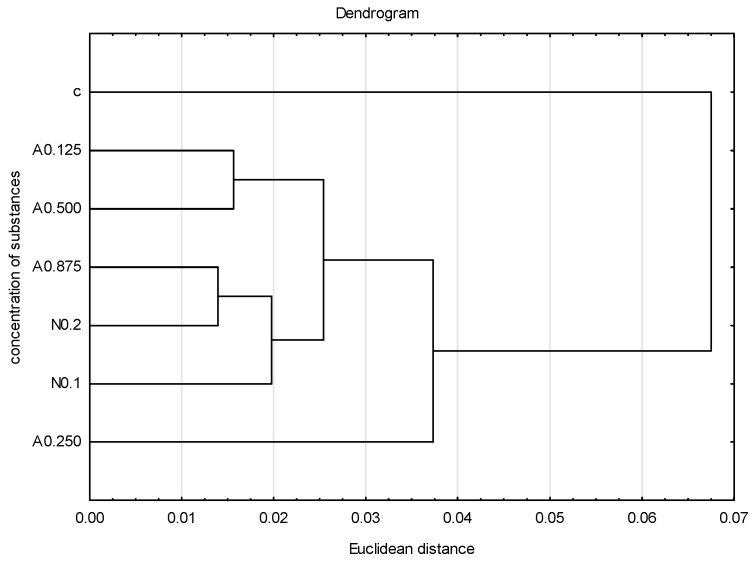
Dendrogram based on five tested veins (A, B, C, E, F) showing the hierarchical relationships between groups. Groups represent different concentrations of acetamiprid (A = acetamipirid: 0.125, 0.250, 0.500, and 0.875 µg/mL), of nicotine (N = nicotine: 0.1 and 0.2 mg/mL), and the control group (c).

To graphically illustrate the obtained results, principal component analysis (PCA) (Figure 4) and cluster analysis (dendrogram) (Figure 5) were performed. Sublethal concentrations of acetamiprid at 0.125 and 0.500 formed one group, and concentrations of acetamiprid at 0.875 and nicotine at 0.2 (which are above SP50 doses) formed the other. The concentrations of acetamiprid at 0.250 and the sample without toxic substances were the most distinct.

**Figure 6:**
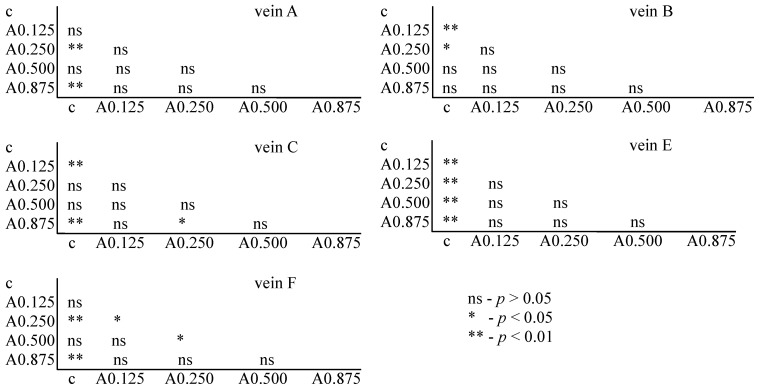
Tukey test for fluctuating asymmetry between tested acetamiprid concentrations (A = acetamiprid: 0.125, 0.250, 0.500, and 0.875 µg/mL) and control (c) for veins A, B, C, E, and F; *p* < 0.01 = statistically significant differences, *p* < 0.005 = statistically significant differences, *p* > 0.05 = differences statistically insignificant (ns).

The Tukey test was performed. The test showed that for acetamiprid, significant differences between the control and FA veins were observed: for vein A at concentrations of 0.250 and 0.875 µg/mL; for vein B at concentrations of 0.125 and 0.250 µg/mL; for vein C at concentrations of 0.125 and 0.875 µg/mL; for vein E at all tested concentrations; and for vein F at concentrations of 0.250 and 0.875 µg/mL. In veins C and F, significant differences were also found between the 0.250 and 0.875 µg/mL concentrations and 0.500 and 0.875 µg/mL concentrations, respectively (Figure 6).

**Figure 7:**
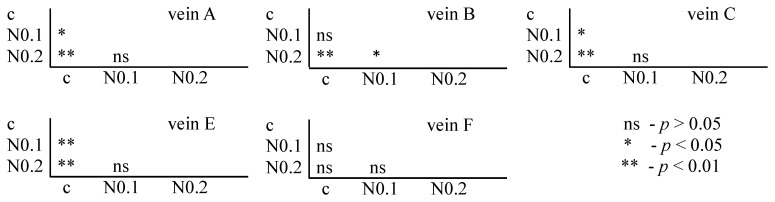
Tukey test for fluctuating asymmetry between tested nicotine concentrations (N = nicotine: 0.1 and 0.2 mg/mL) and control (c) for veins A, B, C, E, and F; *p* < 0.01 = statistically significant differences, *p* < 0.005 = statistically significant differences, *p* > 0.05 = differences statistically insignificant (ns).

For nicotine, the Tukey test showed significant differences between control and FA veins: vein A at concentrations of 0.1 and 0.2 mg/mL; vein B at a concentration of 0.2 mg/mL; and veins C and E at concentrations of 0.1 and 0.2 mg/mL. In vein B, significant differences were also found between the 0.1 and 0.2 mg/mL concentrations (Figure 7).

**Figure 8:**
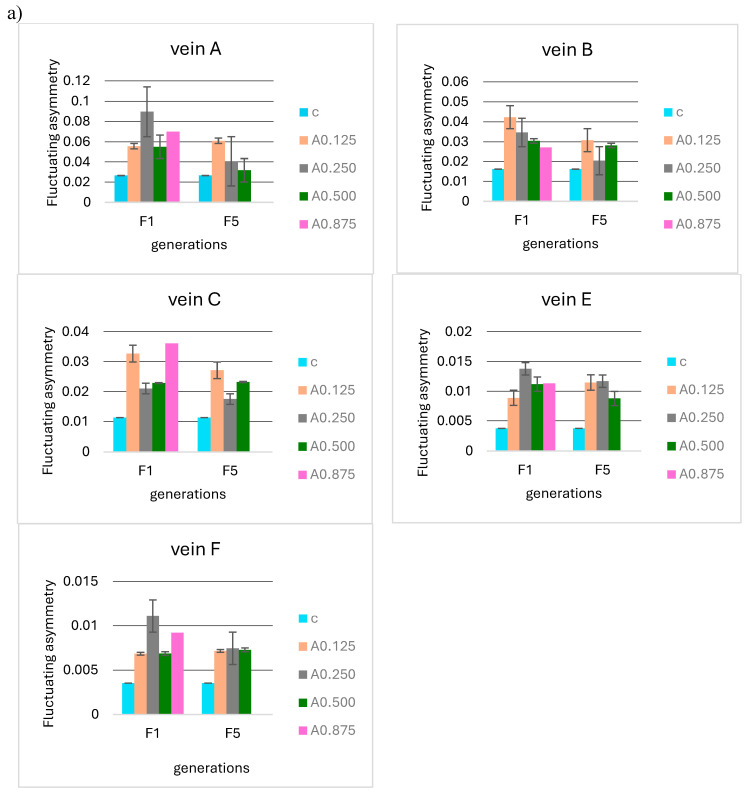

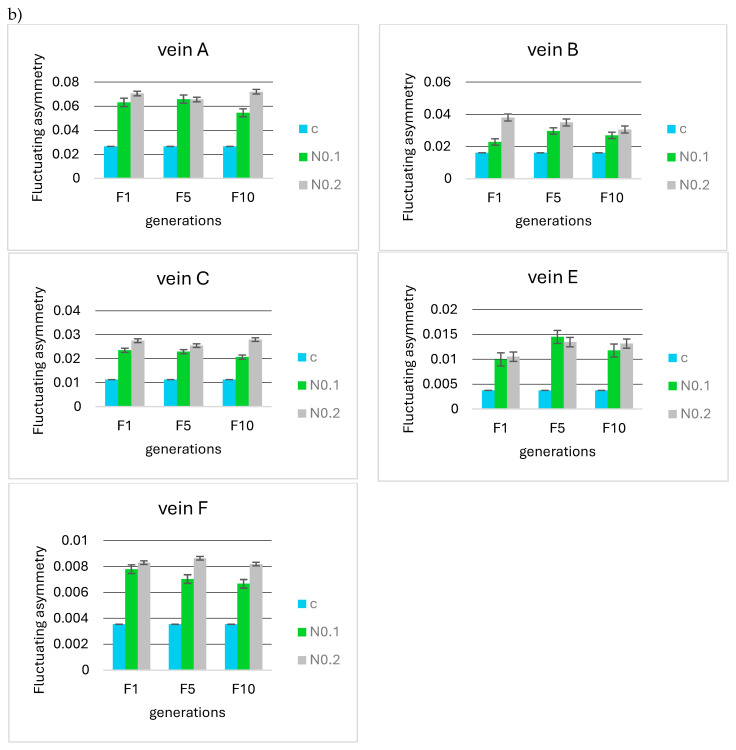
Mean fluctuating asymmetry of veins A, B, C, E, and F observed in successive insect generations (F1 = first generation, F5 = fifth generation, F10 = tenth generation) for the control (c) and (**a**) different concentrations of acetamiprid (A0.125, 0.250, 0.500, 0.875 µg/mL) and (**b**) different concentrations of nicotine (N0.1 and 0.2 mg/mL).

No significant differences were observed when analyzing the F5 and F10 generations in relation to the first generation in terms of the mean FA for 0.125, 0.250, 0.500, and 0.875 µg/mL acetamiprid concentrations (Figure 8a) and 0.1 and 0.2 mg/mL nicotine concentrations (Figure 8b). Fluctuating asymmetry did not increase in subsequent generations and was smaller than in F1 in some cases.

**Figure 9:**
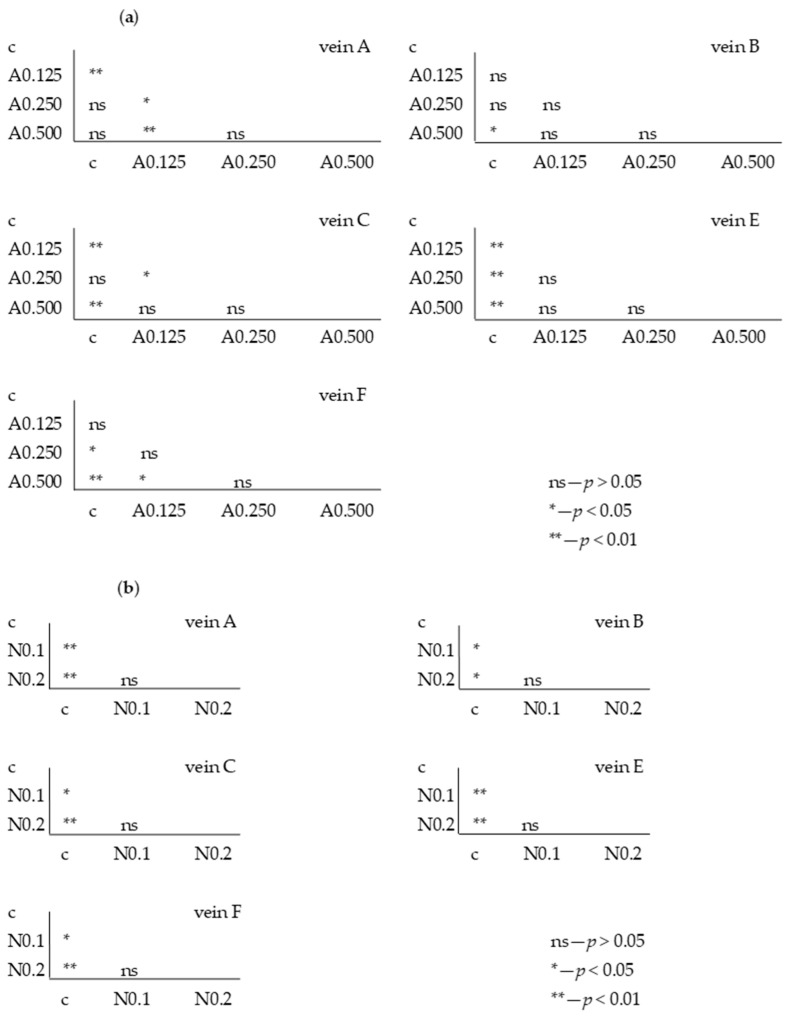
Tukey test for fluctuating asymmetry between control (c) and tested acetamiprid concentrations (A = acetamiprid: 0.125, 0.250, 0.500, 0.875 µg/mL) (**a**) and between control (c) and tested nicotine concentrations (N = nicotine: 0.1 and 0.2 mg/mL) (**b**) for veins A, B, C, E, and F; *p* < 0.01 = statistically significant differences, *p* < 0.005 = statistically significant differences, *p* > 0.05 = differences statistically insignificant (ns); fifth generation.

For the fifth generation, the Tukey test was also performed, showing statistically significant differences for individual veins between the control sample in some acetamiprid and nicotine concentrations (Figure 9a); however, these were not significant between F1 and F5.

For nicotine, the Tukey test showed significant differences between the FA of the control and all tested concentrations (Figure 9b), but these were not significant at the intergenerational level.

## 4. Discussion

Despite the undoubted benefits of reasonable insecticide use, these chemicals can be serious risks to humans or the environment [31]. Neonicotinoids, a new group of insecticides, have become popular in recent years; initially, they were considered a safe alternative to other substances that control pests, but many of them are dangerous and have already been banned. One of the few neonicotinoids currently approved is acetamiprid, a neurotoxin that affects the nervous system of insects and is used to control SWD, an invasive fruit pest. Despite renewed approval from the Commission Implementing Regulation, this substance has been detected in residues in many products, possibly posing previously unrecognized risks [32]. The EFSA recommends updating its residue limits based on new information on the risks involved and continuing research. We decided to use SWD to study the effects of sublethal doses of acetamiprid. Acetamiprid is classified as harmful to SWD parasitoids because it causes rapid mortality and reduced survival time in offspring and does not show parasitism [33]. As a positive control with a documented harmful effect on Drosophila, we used nicotine [23,34]. In earlier reports, we confirmed that in low doses, acetamiprid adversely affects *D. suzukii* development and reproduction, and we were interested in whether it similarly affects another bioindicator of stress, fluctuating asymmetry.

This small, random deviation from ideal symmetry measures developmental instability, a sensitive biomarker of environmental stress [24,25,26,35]. Higher FA has been proved to be a measure of susceptibility to pesticides in, for example, lizards [36], frogs [37], birds [38], fish [39,40], and invertebrates: Odonata [41], Apis [42], Triatoma [43,44], carabid beetles [45], and Drosophila [46,47].

FA in locomotor features may be particularly important in the fitness context; therefore, we decided to study wings—or more precisely, their veins—to determine whether long-term exposure to various sublethal acetamiprid and nicotine concentrations throughout the developmental period would cause asymmetry. Studies conducted on *D. melanogaster* have shown FA in wings under the influence of the antibiotic neomycin [27], but not all veins have shown FA, and it has not always been precisely correlated with increased antibiotic concentrations. In our studies, five of the six analyzed veins showed differences between the right and left wings under the influence of sublethal doses of insecticides, classified as FA after statistical analysis. Compared with the control, the differences were statistically significant and correlated with increased insecticide concentrations in most cases. The average wing vein length was smaller in the insects exposed to toxins than in the controls. Similar results have been reported for *D. melanogaster* by [27,30,48]. Their studies showed that both acetamiprid and nicotine, even in exceedingly small doses, are a strong controlling factor for SWD, disturbing developmental stability. Importantly, we can relate the results of our analyses to other studies on the effects of the same concentrations of acetamiprid and nicotine [23] and link the appearance of FA to locomotor dysfunction occurring under the influence of the tested toxins. This is a significant result. In subsequent generations of SWD, we did not observe major differences in the FA index. The results were similar, possibly indicating that insects do not acquire resistance to this pesticide. The reduced number of flies in subsequent generations indicates high acetamiprid activity even at low doses; with each subsequent generation, the number of insects exposed to insecticides decreased compared with the control culture. Acetamiprid had a greater effect on the decreasing numbers than nicotine used in higher concentrations. This also shows the validity of using FA as an environmental stress indicator. Exposure to low doses of insecticide and their influence on insects have been observed in many different experiments. Among sublethal effects, one can mention modification of antioxidant enzymes, altered neurotransmission, especially inhibited acetylcholinesterase activity, or structural aberrations [49]. Research on nicotine showed that sublethal effects, like reduced vitality or fertility, due to physiological malfunctions and structural malformations, may significantly decrease the fitness of flies [30]. A major problem with the widespread use of insecticides is the emergence of resistance. This enlarges the toxic effects of pesticides on the environment and leads to an increase in the amount of insecticides required to effectively control resistant insects. In the case of acetamiprid, we did not observe the acquisition of resistance expressed in fluctuating asymmetry. On the one hand, this confirms the effectiveness of this pesticide in controlling SWD even when used at low doses over several generations. On the other hand, attention should be paid to the risk of harmful effects of low doses in case of interactions with non-target organisms, including humans. This is especially true since small amounts of residues of this compound can be found not only in the environment but also in food.

## 5. Conclusions

The conclusions from our research are as follows:

- Sublethal doses of acetamiprid and nicotine disrupt the developmental stability of *Drosophila suzukii*, which manifests as fluctuating asymmetry in wing veins.
- Exposing insects to these insecticides throughout development reduces wing vein length, consequently reducing wing size.
- It is likely that persistent FA in subsequent SWD generations affects increased mortality—assuming that more disturbed individuals do not develop from eggs or larvae.
- FA can be used to analyze the effects of acetamiprid even at low, sublethal concentrations.

## Figures and Tables

**Table 1:** Descriptive statistics for five studied veins in *Drosophila suzukii* wings. Yellow indicates the highest average vein length, and green indicates the lowest.

Vein	Concentration: A = Acetamiprid [µg/mL] N = Nicotine [mg/mL]	N	Mean [mm]	Min [mm]	Max [mm]	SD	V
A	C	270	64.41	54.42	75.14	5.624	8.724
	A0.125	180	57.62	46.71	81.34	8.005	13.89
	A0.250	180	67.81	52.97	75.88	5.597	8.817
	A0.500	180	69.92	60.66	82.705	5.508	7.877
	A0.875	90	66.02	56.13	76.47	6.653	10.077
	N0.1	270	60.23	49.53	68.67	5.21	8.64
	N0.2	270	62.35	50.03	70.56	4.53	7.27
B	C	270	195.07	166.13	225.26	17.038	8.734
	A0.125	180	183.48	151.61	232.45	23.92	13.032
	A0.250	180	189.47	170.64	207.69	11.771	6.032
	A0.500	180	194.72	148.17	218.5	15.391	7.904
	A0.875	90	187.81	160.29	217.05	14.741	7.85
	N0.1	270	170.32	146.49	195.92	12.83	7.53
	N0.2	270	173.44	153.87	192.72	12.62	7.28
C	C	270	357.88	328.29	389.47	15.687	4.383
	A0.125	180	340.27	297.4	392.24	27.21	8.00
	A0.250	180	340.63	295.05	373.29	24.116	7.12
	A0.500	180	351.12	304.88	382.38	21.01	5.98
	A0.875	90	329.92	285.17	368.57	18.63	5.65
	N0.1	270	315.81	285.03	354.42	19.59	6.20
	N0.2	270	310.302	281.32	343.97	18.17	5.85
D	C	270	539.19	480.89	590.16	33.81	6.27
	A0.125	180	499.42	436.04	586.77	39.76	7.96
	A0.250	180	513.65	457.61	557.28	31.27	6.09
	A0.500	180	519.2	433.63	570.6	32.7	6.30
	A0.875	90	506.02	435.86	545.57	27.97	5.53
	N0.1	270	501.28	446.47	560.87	34.42	6.87
	N0.2	270	495.16	440.85	549.49	34.94	7.06
E	C	270	1145.43	1059.64	1244.9	65.01	5.68
	A0.125	180	1098.38	974.57	1264.04	73.99	6.74
	A0.250	180	1073.35	930.79	2272.98	65.58	6.11
	A0.500	180	1118.23	925.57	1231.3	755.5	6.75
	A0.875	90	1067.16	999.2	1210.04	66.31	6.21
	N0.1	270	1005.16	890.91	1114.17	64.98	6.46
	N0.2	270	1009.78	930.77	1106.28	60.45	5.99
F	C	270	1754.62	1609.57	1893.18	98.64	5.62
	A0.125	180	1676.69	1500.75	1936.74	120.99	7.22
	A0.250	180	1663.93	1523.23	1783.37	91.16	5.48
	A0.500	180	1718.81	1428.33	1886.32	108.53	6.31
	A0.875	90	1653.04	1487.69	1823.82	95.06	5.75
	N0.1	270	1580.19	1387.73	1753.63	102.91	6.51
	N0.2	270	1579.43	1447.36	1714.62	98.12	6.21

N = number of measurements; Min = minimum value; Max = maximum value; SD = standard deviation; V = coefficient of variation; 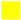 = highest average vein length; 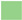 = lowest average vein length.

**Table 2:** Descriptive statistics for fluctuating asymmetry in five examined F1 *Drosophila suzukii* wing veins. Blue indicates the highest mean FA observed for acetamiprid, and green indicates the highest mean FA observed for nicotine.

Vein	Concentration: A = Acetamiprid [µg/mL] N = Nicotine [mg/mL]	N	Mean [mm]	Min [mm]	Max [mm]	SD	V
A	C	30	0.026709	0.001671	0.086230	0.025	94.83
	A0.125	30	0.055673	0.004383	0.182970	0.049	88.16
	A0.250	30	0.089643	0.014400	0.225400	0.057	63.65
	A0.500	30	0.054924	0.003820	0.117420	0.035	63.10
	A0.875	30	0.069924	0.004559	0.271510	0.073	104.27
	N0.1	30	0.063087	0.001311	0.300860	0.058	92.16
	N0.2	30	0.070425	0.001700	0.206100	0.058	82.07
B	C	30	0.016205	0.001827	0.050997	0.013	82.03
	A0.125	30	0.042344	0.011310	0.095230	0.029	69.14
	A0.250	30	0.034723	0.005800	0.134000	0.025	72.19
	A0.500	30	0.030442	0.003770	0.090200	0.020	64.65
	A0.875	30	0.027121	0.001325	0.090630	0.022	81.17
	N0.1	30	0.022847	0.001152	0.059800	0.016	70.68
	N0.2	30	0.038047	0.000950	0.105520	0.027	71.08
C	C	30	0.011279	0.001560	0.048240	0.009	81.71
	A0.125	30	0.032707	0.002972	0.092920	0.023	71.51
	A0.250	30	0.021047	0.001500	0.049600	0.014	68.11
	A0.500	30	0.022863	0.006325	0.049450	0.012	55.04
	A0.875	30	0.036076	0.001990	0.102430	0.029	81.45
	N0.1	30	0.023521	0.001485	0.060170	0.019	81.48
	N0.2	30	0.027501	0.000580	0.097310	0.023	83.49
E	C	30	0.003732	0.001014	0.007978	0.002	52.22
	A0.125	30	0.008884	0.001504	0.034520	0.008	89.21
	A0.250	30	0.013779	0.001190	0.059710	0.011	83.41
	A0.500	30	0.011182	0.001870	0.031630	0.006	56.72
	A0.875	30	0.011309	0.001350	0.049930	0.011	97.76
	N0.1	30	0.009978	0.001190	0.044610	0.008	85.28
	N0.2	30	0.010514	0.000380	0.026540	0.009	83.92
F	C	30	0.003535	0.001005	0.009060	0.002	70.37
	A0.125	30	0.006564	0.001660	0.023770	0.005	77.08
	A0.250	30	0.011096	0.001425	0.032600	0.008	76.78
	A0.500	30	0.006859	0.001775	0.017720	0.004	56.61
	A0.875	30	0.009213	0.001373	0.037380	0.007	77.88
	N0.1	30	0.017789	0.000030	0.333850	0.060	336.69
	N0.2	30	0.008290	0.001303	0.026690	0.006	69.59

N = number of measurements; Min = minimum value; Max = maximum value; SD = standard deviation; V = coefficient of variation; 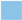 = highest mean FA observed for acetamiprid; 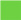 = highest mean FA observed for nicotine.

## Data Availability

The data presented in this study are available upon request from the corresponding author.
